# Integrin-α9β1 as a Novel Therapeutic Target for Refractory Diseases: Recent Progress and Insights

**DOI:** 10.3389/fimmu.2021.638400

**Published:** 2021-03-15

**Authors:** Shihan Xu, Tingwei Zhang, Zhengguo Cao, Wenjie Zhong, Chuangwei Zhang, Han Li, Jinlin Song

**Affiliations:** ^1^ College of Stomatology, Chongqing Medical University, Chongqing, China; ^2^ Chongqing Key Laboratory of Oral Diseases and Biomedical Sciences, Chongqing, China; ^3^ Chongqing Municipal Key Laboratory of Oral Biomedical Engineering of Higher Education, Chongqing, China; ^4^ The State Key Laboratory Breeding Base of Basic Science of Stomatology (Hubei-MOST) & Key Laboratory of Oral Biomedicine Ministry of Education, School & Hospital of Stomatology, Wuhan University, Wuhan, China; ^5^ Department of Periodontology, School & Hospital of Stomatology, Wuhan University, Wuhan, China

**Keywords:** integrin-α9β1, cancer, autoimmune diseases, axon regeneration, thrombosis

## Abstract

Integrins refer to heterodimers consisting of subunits α and β. They serve as receptors on cell membranes and interact with extracellular ligands to mediate intracellular molecular signals. One of the least-studied members of the integrin family is integrin-α9β1, which is widely distributed in various human tissues and organs. Integrin-α9β1 regulates the physiological state of cells through a variety of complex signaling pathways to participate in the specific pathological processes of some intractable diseases. In recent years, an increasing amount of research has focused on the role of α9β1 in the molecular mechanisms of different refractory diseases and its promising potential as a therapeutic target. Accordingly, this review introduces and summarizes recent research related to integrin-α9β1, describes the synergistic functions of α9β1 and its corresponding ligands in cancer, autoimmune diseases, nerve injury and thrombosis and, more importantly, highlights the potential of α9β1 as a distinctive target for the treatment of these intractable diseases.

## Introduction

Integrins are specific transmembrane proteins that function as receptors on the surface of cell membranes. The heterodimers of integrin members formed by noncovalent bonds of α-subunits and β-subunits in response to corresponding ligands in the extracellular matrix mediate intracellular mechanical and chemical signals (1). So far, 24 distinct αβ receptor complexes composed of 18 α-subunits and 8 β-subunits have been found, which play regulatory roles in different developmental and physiological processes (2, 3). However, within the integrin family, there has been relatively little research on α9β1.

Integrin-α9 (ITGA9) was found in guinea pig airway epithelial cells in 1991, when the polymerase chain reaction (PCR) technique showed a new α-subunit with a novel sequence compared to previously reported integrin subunits (4). In 1993, the human amino acid sequence and cDNA of the new subunit was determined and definitively designated as α9 (5). The α9-subunit specifically groups together with the β1-subunit, combining to form the unique heterodimer integrin-α9β1, which acts as an indispensable receptor for cellular signal responses (6).

In the last few decades, more and more research has focused on the roles of integrin-α9β1 in periods of growth, development and disease. More importantly, integrin-α9β1 has been reported as a new therapeutic target for some specific refractory diseases.

In this review, we focus on recent advances in research on integrin-α9β1. We will discuss its individual potential clinical value for the treatment of tumors, rheumatoid arthritis (RA), axon damage and thrombosis.

## Structure and Function of Integrin-α9β1

The *ITGA9* gene is distributed on the human chromosome 3p21.3-22.2 segment, encodes the polypeptides of 1035 amino acids, and has a size of 114.5 KD (7). The structure of α9 consists of three parts: a large N-terminal extracellular domain, a transmembrane segment and a short C-terminal cytoplasmic tail (8). Among this structure, the N-terminal portion mediates ligand binding (9), while the cytoplasmic domain specifically binds to intracellular proteins that modulate the physiological activity of cells, such as spermidine/spermine N1-acetyltransferase, which has been proven to regulate inward rectification of the inward-rectifier K+ channel to enhance cell migration with interaction of the α9 cytoplasmic domain (10).

According to their homology, α-subunits can be divided into three families. One includes subunits with a characteristic disulfide-linked cleavage site and forms heterodimers that recognize Arg-Gly-Asp (RGD)-containing ligands. Another includes subunits that contain an inserted domain close to the N-terminus but no cleavage site, which generally do not recognize RGD-containing ligands. However, α9 and α4 can form a special third subfamily that contains neither the insertion domain nor the disulfide-linked cleavage site and does not recognize the classic integrin-binding motif RGD (11). The subfamily is one of the newest and most specific integrin families from an evolutionary perspective and is only expressed in vertebrates (12). Integrin-α9 used to be known as ITGA4L (integrin-α4-like) since α9 and α4 show peptide sequence similarities (39% amino acid identity) and share several common ligands (13, 14). However, knockout of α9 and α4 results in different phenotypes in mice: α4-knockout mice die by 11-14 embryonic days due to improper chorioallantoic fusion, cardiac hemorrhage (15), and defects in all hematopoietic lineages in the fetal liver, bone marrow, and spleen (16); while α9-knockout mice die within 12 days of birth with bilateral chylothorax (17), impaired development of neutrophils (18), and defective development of lymphatic valves and venous valves (19). This indicates that α9 and α4 exert distinct as well as similar physiological functions *in vivo*. In addition, it is intriguing that the integrin family extensively regulates cellular directional migration in an electric field (galvanotaxis), which is necessary for precise control of wound healing, angiogenesis, immune responses and organismal development. In this potentially endogenous guidance mechanism, cells expressing α9 migrate to an anode in an electric field, whereas cells expressing α4 migrate in random directions in an electric field (20), demonstrated that integrin-α9 and α4 may differentially contribute to cell migration *in vivo*.

In mouse tissue, immunohistochemistry has shown that integrin-α9β1 is located in the epithelia and muscle of the trachea, digestive system, skin, veins, liver and spleen, but not in the aorta, pancreas or heart (5). Integrin-α9β1 plays an important role in cell adhesion and migration, but more and more research is showing that it has roles far beyond that. It binds to a diversity of ligands in the extracellular matrix, like the a-disintegrin and metalloprotease (ADAM) family, elastic microfibril interface-located protein1 (EMILIN1), vascular endothelial growth factor (VEGF), the extra domain A (EDA) of fibronectin, tenascin-C (TNC), osteopontin (OPN), vascular cell adhesion molecule-1 (VCAM-1) and C-motif-ligand-1 (XCL1)/lymphotactin (21–25). The interaction between these proteins and integrin-α9β1 is vital for organismal growth and development and cellular physiological activities (**Table 1**). Recent studies have focused on the role of α9β1 in pathological processes and its potential as a therapeutic target. And great progress has been made towards certain specific refractory diseases, including various malignant tumors, autoimmune diseases, nerve damage and thrombotic diseases. Here, we will describe in detail how integrin-α9β1 plays a pivotal role in these diseases and, more importantly, makes a promising target for clinical treatment.

**Table 1:** Major ligands and functions interacting with integrin-α9β1.

Ligands	Functions	References
ADAM1, 2, 3, 7, 9, 15, 28	Cell adhesion	(21, 26–28)
ADAM8	Cell adhesion; stimulates osteoclast differentiation	(14, 29)
ADAM12	Cell adhesion; promotes myoblast fusion	(14, 30)
ADAM33	Cell adhesion; involved in asthma pathogenesis	(14, 31)
EMILIN1	Inhibits dermal fibroblast and keratinocyte proliferation; anti-proliferation; essential for lymphatic valve formation and maintenance.	(22, 32–34)
VEGF-A	Cell migration/adhesion; angiogenesis	(35)
VEGF-C	Cell migration/adhesion; promotes sprouting of lymphatics	(23, 36, 37)
VEGF-D	Cell migration/adhesion; promotes sprouting of lymphatics	(23, 36, 38)
EDA of fibronectin	Cell migration/adhesion; promotes filopodia formation; required for lymphatic valve morphogenesis; induces epithelial-mesenchymal transition (EMT); sustains subpopulation of CD133^+^/CD44^+^ cancer cells	(39–43)
TNC	Cell migration/adhesion; cell proliferation; participates in wound healing, fibrosis and neovascularization; participates in neuronal regeneration; required in the bone marrow microenvironment primed for hematopoietic regeneration; mediates inflammatory response	(25, 44–48)
VCAM-1	Cell migration/adhesion; regulates lymphatic development	(49, 50)
OPN	Cell migration/adhesion/chemotaxis; participates in wound healing, fibrosis and neovascularization; mediates inflammatory response; critically involved in the exacerbation of liver fibrosis; contributes to tumor growth and angiogenesis	(25, 51–55)
XCL1/lymphotactin	Cell migration; mediates inflammatory response	(56)
Thrombospondin-1	Cell migration; promotes angiogenesis	(57)
Blood coagulation factor XIII	Cell adhesion	(58)
L1-cell adhesion molecule	Cell adhesion	(59)
Nerve growth factor	Cell chemotaxis and proliferation	(60)
Propolypeptide of von Willebrand factor	Cell adhesion	(58)
Tissue transglutaminase	Cell adhesion	(58)
Plasmin	Cell migration	(61)

### Integrin-α9β1 and Corresponding Ligands Have Potential as Tumor Therapeutic Targets

Integrin-α9 is considered to be closely related to the growth, metastasis and tissue invasion of cancer. The tumor microenvironment provides a large number of ligands in the extracellular matrix, leading to complex and diverse reactions with integrins (62). The expression of α9β1 is up-regulated in many cancers and affects tumor progression through a variety of mechanisms, including regulation of cell migration and invasion, mediation of cell cycle regulatory elements, promotion of the growth of tumor-associated blood and lymphatic vessels, and alternation of the epithelial-mesenchymal transition [EMT; (23, 35, 63, 64)]. Hence, there have been successive reports on the performance of α9β1 in cooperating with specific ligands in different cancers, and on the experimental effects of ITGA9-targeted therapeutic agents (**Table 2**).

**Table 2:** Overview of experimental trials with therapeutic agents targeting ITGA9 in different cancers.

Types of cancer cells	Function of integrin-α9β1	Involved ligands	Participating mechanism and signaling pathways	Therapeutic agent targeting ITGA9	References
VCaP (prostate cancer metastatic cell line)	Promotes bone metastasis of prostate cancer	TNC		mAb Y9A2; siRNA	(44)
G361 (human melanoma cells)	High activation (activated by Mn^2+^) induces cell focal adhesions	TNC; ADAM-12	Through Rho kinase pathway and vesicle exocytosis	mAb Y9A2; Primaquine (inhibitor of vesicle trafficking)	(65)
G361	Normally supports cell migration	TNC; ADAM-2, -3, -12, -15	Through GTPase Rac signaling and vesicle exocytosis	mAb Y9A2; Primaquine; VLO5	(26, 65, 66)
RAW264.7 (mouse macrophage line)	Enhances angiogenesis, melanoma growth and migration	OPN	Up-regulates COX-2, PGE_2_ and MMP-9 through the p38 and ERK signalling pathways	siRNA	(51)
Mel Ju and Mel Im (cutaneous malignant melanoma cell lines)	Promotes the EMT and cell invasion		Induces the expressions of mesenchymal markers: vimentin, SNAIL and N-cadherin	siRNA; miR-125b	(67)
A375 and A875 (melanoma cell lines)	Promotes cell proliferation, migration, glucose consumption, lactate production, EMT and inhibits apoptosis		Up-regulates hexokinase 2 (HK2), proliferating cell nuclear antigen (PCNA), cyclin D1 and B-cell lymphoma (Bcl)-2	MiR-296-3p; si- and sh-CCAT1	(68)
LM2 and SUM159 (triple-negative breast cancer cell lines)	Associates with cancer stem cell-like property, tumor angiogenesis, growth and metastasis		ITGA9 depletion promotes β-catenin degradation through the ILK/PKA/GSK3 pathway and affects the Wnt/β-catenin pathway	siRNA	(69)
468LN (a variant of the 468GFP human breast cancer cell line)	Involves in migratory and invasive functions. Obligatory for promoting tumor-associated lymphangiogenesis and lymphatic metastasis.	VEGF-C; VEGF-D	Activates ERK signalling pathway	mAb sc-59969; siRNA	(38)
LLC-1 (Lewis lung carcinoma cells); SW480 (colon carcinoma cells)	Induces tumor growth, vasculogenesis and metastasis. Promotes molecular and cytoskeletal changes consistent with EMT.	TNC	Induces phosphorylation of Src-Y416 and β-catenin-Y654; forms a tri-partite complex with E-cadherin and β-catenin, which dissociates following α9β1 interaction with ligands	mAb Y9A2; VLO5; si- and sh- RNA	(64, 70, 71)
Colorectal carcinomas cells; A549 (human lung cancer cell line); NCI-H522 (human lung adenocarcinoma cell line)	Promotes metastatic capacity, EMT and invasion. Leads to increased mesenchymal markers and decreased epithelial markers.	Fibronectin-EDA	Activates FAK/c-Src and MEK/ERK signalling pathways and activates the small GTPase Rac1	mAb; Irigenin	(39, 72)
SW480; HMVECs (human dermal microvascular endothelial cells)	Supports SW480 cell adhesion, migration and invasion. Supports HMVEC in forming the endothelial tube.	VEGF-A (EYP peptide)	Activates the integrin signaling intermediates Src and FAK	mAb Y9A2; siRNA	(73)
SW480	Significantly elevates and is essential for CD133/CD44-positive cells. Promotes spheroid formation. Inhibits cell apoptosis.	Fibronectin-EDA	Activates FAK/ERK and sustains the Wnt/β-catenin signaling pathway; blocking of α9β1 up-regulates cleaved caspase 3, 9, cleaved poly (ADP-ribose) polymerase with suppression of cyclin D1	mAb Y9A2	(40)
RH30, CW9019 and HTB-82 (rhabdomyosarcoma cell lines)	Promotes cell adhesion, motility and invasion		Notch pathway mediates the expression of ITGA9 through downstream effectors NICD and Hes1	mAb 3E4; GSI (Notch signaling inhibitor)	(74)
Rhabdomyosarcoma cell lines RD (embryonal subtype) and CW9019 (alveolar subtype)	Supports cell proliferation and cell invasiveness		Activates FAK signaling pathway	miR-7; miR-324-5p; sh-RNA	(75)
OSCC13 cell line; CD11c+ myeloid cells; lymphatic endothelial cells	Increases expression of CCL21. Orchestrates an immune-suppressive microenvironment to facilitate tumor escape from immunosurveillance.	TNC	Supports CCL21/CCR7 signalling pathway; Regulate the crosstalk and position of tumor-infiltrating lymphocytes like CD11c+ cells and Tregs	Ab; BOP (an antagonist for integrin-α9β1)	(76)
SMMC-7721 and MHCC-LM3 (hepatocellular carcinoma cell lines)	Prevents tumor cell migration and invasiveness *in vitro*, and tumor growth and metastasis *in vivo*.		Suppresses the FAK/Src-Rac1/RhoA signaling pathway and disrupts focal adhesion reorganization	Lentivirus transduction of human ITGA9 ORF subclone	(77)

#### Prostate Cancer

The NotI-microarray analysis has identified *ITGA9* as a potential biomarker for the detection and discrimination of prostate tumors with different aggressiveness and malignancy (78). In a study of prostate cancer, a human bone metastasis tissue array consisting of 63 metastasis samples was analyzed. The cells showed immunoreactivity to *ITGA9* in 74% of the cancer foci associated with TNC, with the latter being an important ligand of integrin-α9β1 (44). In prostate cancer, the deposition of TNC during early cancer progression is a key marker of stromal microenvironment alternation, which is overexpressed in the endosteum when normal processes of cellular senescence and death lead to microfracture repair (79, 80). Then, bone metastatic cells interact with TNC in the endometrium through the integrin-α9β1 to accelerate the spread, whereas the activity is eliminated by small interfering RNA (siRNA) or neutralizing antibodies of α9 (44). Besides, the reaction site where human TNC binds to α9β1 is located in the isoleucine-aspartic acid-glycine motif within the third fibronectin type III repeat, which is absent in mice, which may explain why cancer rarely metastasizes to the bone in murine models, while both integrin-α9β1 and TNC are thought to have great significance in metastatic prostate cancer (81, 82).

#### Melanoma

**Figure 1:**
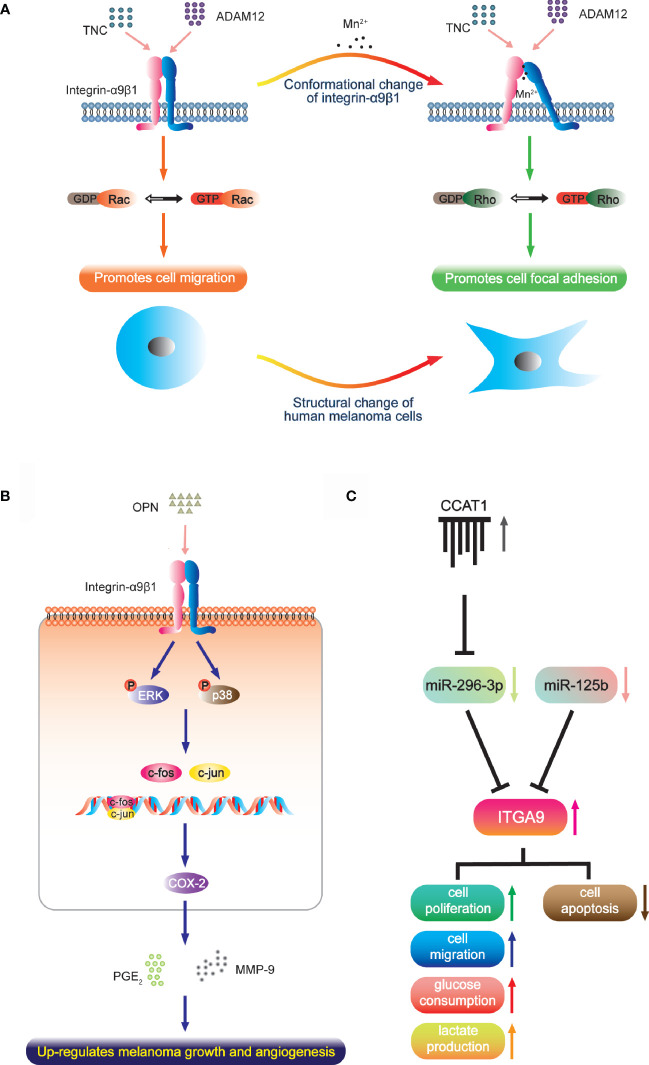
The function of integrin-α9β1 in melanoma. **(A)** Under normal conditions, the intermediate activity state of integrin-α9β1 supports cell migration *via* interaction with TNC and ADAM12. A high activation state (converted by manganese ions, which occur at much higher levels in melanoma than in other cancers) changes the integrin conformation and cell morphology, and induces and localizes to focal adhesions. **(B)** In tumour-associated macrophages, integrin-α9β1 binds to OPN, activating ERK- and p38-dependent AP-1, ultimately leading to enhanced expressions of COX-2, PGE2 and MMP-9, which contribute to melanoma growth and angiogenesis. **(C)** miR-296-3p (regulated by lncRNA CCAT1) and miR-125b directly target ITGA9 to mediate the cell physiology of melanoma.

Melanoma has high aggressiveness and metastasis, which has been defined as *lethal melanocytic neoplasm* (83). Integrin-α9β1 is up-regulated in melanoma tissue and cells, and different active states produce different effects. Under normal circumstances, the intermediate activity state of α9β1 supports cell migration through interaction with TNC and ADAM12, which are regulated by guanosine triphosphatase (GTP)-Rac signaling. However, manganese ions stimulate highly-active transformation with a protein conformation change of α9β1, which then leads to cell focal adhesion accompanied by morphological change that is dependent on the Rho kinase pathway [**Figure 1A**; (65, 84)]. It has been reported that focal adhesion in tumor cell is closely associated with resistance to radio- and chemotherapy. Considering that melanoma has much higher manganese levels than other cancers, this mechanism may explain the extreme radio- and chemo-resistant nature of melanoma and its low patient survival (85, 86). Another study reported that integrin-α9β1 binds to the OPN-activating p38- and ERK-signaling pathways and activator protein (AP)-1. This ultimately leads to expression of cyclooxygenase-2 (COX-2) and accompanying secretion of prostaglandin E_2_ (PGE_2_) and matrix metalloproteinase (MMP)-9 in tumor-associated macrophages (**Figure 1B**). These factors contribute to melanoma growth and angiogenesis (51).

The function-blocking anti-human α9 monoclonal antibody Y9A2 has the ability to inhibit adhesion of human melanoma cells. So does VLO5, a snake disintegrin that antagonizes α9β1 (26, 66). Furthermore, the regulation of endogenous integrin-α9β1 depends on cell vesicle exocytosis, and the use of primaquine (an effective inhibitor of vesicle trafficking) effectively attenuates melanoma cell attachment in a dose-dependent manner (65). These results reveal the effect of anti-α9 treatment. Moreover, *ITGA9* has been reported to be the direct target of miR-125b and miR-296-3p. The expression of miR-125b is clearly decreased in primary melanoma, and even less so in the metastatic invasion phenotype, which is negatively correlated with the expression of *ITGA9*. Integrin-α9β1 advances cancer growth and metastasis by potentiating EMT (64) so that miR-125b shows the capacity to inhibit malignant melanoma cell invasion and metastasis by targeting *ITGA9 in vitro* and *in vivo* (67). Similarly, miR-296-3p is also downregulated in melanoma cells and tissue. It targets *ITGA9* to inhibit glucose metabolism, lactic acid production, proliferation and migration of melanoma cells while inducing cell apoptosis. In addition, the long noncoding RNA (lncRNA) CCAT1 acts as a competing endogenous RNA that sponges miR-296-3p to up-regulate *ITGA9 in vivo*; thus, CCAT1 silencing inhibits melanoma cell growth (68). This field of research provides a new direction for the treatment of melanoma with micro-RNA and lncRNA that target *ITGA9* either directly or indirectly (**Figure 1C**).

#### Breast Cancer

Integrin-α9 exists in normal human breast tissue, but the expression levels are heterogeneous in breast tumors (87). In a study of 38 human samples, the mRNA expression level was normal or increased in 45% of tumors, but decreased or absent in another 44% of samples, while 11% of samples showed that *ITGA9* had a mutation or pathological alternative splicing (88). Hypermethylation of CpG-island is considered to be the critical mediation mechanism of *ITGA9* inactivation of breast malignant tumors. In triple-negative breast cancer, the level of *ITGA9* is drastically higher than in other subtypes of breast cancer, which is related to worse distant metastasis-free survival and recurrence-free survival rates. The nanoparticle-mediated delivery of *ITGA9* siRNA into tumor cells has the capacity to sharply down-regulate angiogenesis, growth and metastasis by inducing β-catenin degradation *via* the integrin-linked kinase (ILK)/protein kinase A (PKA)/glycogen synthase kinase 3 (GSK3) pathway (69). The Wnt/β-catenin pathway is a crucial cascade involved in the development of cancer and is linked to decreased overall survival in breast cancer patients (89, 90). Knockdown of *ITGA9* promotes β-catenin degradation, suggesting that integrin-α9 may interfere with the Wnt signaling pathway to influence the tumor microenvironment. On the other hand, integrin-α9β1 interacts with VEGF-C and -D (produced by cancer cells or by macrophages) to confer the functions of migration and invasiveness in human breast cancer cell line 468LN, which can be abrogated by antibody blocking or stable knockdown of integrin-α9. Similarly, the knockdown of *ITGA9* abrogates primary tumor growth, angiogenesis, metastatic ability to the draining lymph node and intra-tumoral lymphangiogenesis *in vivo* in nude mice (38).

#### Lung Cancer

In human primary small cell lung cancer, the long-term survival rate of patients is significantly lower with higher expression of α9β1. Injection of LLC-1 lung carcinoma cells or SW480 colon carcinoma cells with over-expression of α9β1 both induce greater tumor growth and metastasis in mice (64). It is worth noting that EMT is considered to be very crucial in metastatic progression, which causes disruption to intercellular contacts and enhances cell motility to release cancer cells from the parent epithelial tissue (91). While α9β1 binding to TNC supports phenotypic and functional alterations to EMT, with increases in N-cadherin, α-SMA, vimentin and snail (mesenchymal markers), as well as a decrease in E-cadherin [epithelial marker; (64)]. The above process is accompanied by phosphorylation of β-catenin through the Src signaling pathway, which has been proved to be closely associated with EMT (70, 71). In addition, fibronectin-EDA also imparts the EMT phenotype through integrin-α9β1 (39), and irigenin (a novel lead from the Western Himalayan chemiome that can be isolated from the rhizomes of *Belamcanda chinensis*) has an anti-metastasis capacity by selectively blocking the α9β1 and α4β1 integrin binding sites with the C-C loop of EDA (92). In human lung cancer cell line A549 and lung adenocarcinoma cell line NCI-H522, irigenin conquers fibronectin-EDA-induced cell proliferation, migration and invasion with dose-dependent inhibition of EMT markers. Hence, irigenin may become a lead compound in the management of lung carcinoma (72).

#### Colon Cancer

*ITGA9* antigen has been detected in the basolateral domain of colonic glandular epithelial cells at the fetal stage, but not in adults under normal circumstances. Comparatively, colon adenocarcinoma cells have the potential to express integrin-α9 with polarization features, thereby the α9 subunit may be conditional on oncofetal pattern expression in the human colonic epithelium (93). The peptide EYP of VEGF-A specifically binds to α9β1 and induces invasion of colorectal cancer cell line SW480 with phosphorylation of the integrin signaling intermediates, Src and focal adhesion kinase [FAK; (73)]. Additionally, endothelial cell-derived fibronectin-EDA binds to α9β1, which promotes colorectal cancer metastasis with EMT phenotypic conversion through activation of the FAK, ERK and Rac signaling pathways and supports endothelial tube formation (39). This research illustrates that cooperation between integrin-α9β1 and paracrine or autocrine extracellular matrix ligands is critical to the colon cancer microenvironment. Furthermore, long-term application of methotrexate, an important drug widely used in cancer therapy, carries the risk of resistance emergence (94). Simultaneously, *ITGA9* is obviously up-regulated in already-developed drug-resistant colon cancer cells, whereas α9 is reported to be a promising target candidate for overcoming methotrexate resistance in colon cancer (95).

CD133^+^/CD44^+^ cancer cells, which have the properties of tumour progenitor cells, are critical in the tumorigenesis of colorectal cancer (96, 97). The expression level of integrin-α9β1 is greatly up-regulated in human colorectal cancer samples and is also increased in the CD133^+^/CD44^+^ subpopulation of SW480 cells compared with the CD133^-^/CD44^-^ subpopulation. Fibronectin-EDA also increases in CD133^+^/CD44^+^ cells and activates the integrin-α9/FAK/ERK pathway to sustain Wnt/β-catenin signaling activity. The latter is critical in the development and progression of colon cancer (40). Blocking of fibronectin-EDA and α9β1 suppresses the clonogenicity and sphere-formation capacity of CD133^+^/CD44^+^ cells, and the monoclonal antibody (mAb) of integrin-α9β1 obviously promotes cell apoptosis by up-regulation of apoptotic protein markers, like caspase-3, caspase-9 and poly (ADP-ribose) polymerase, concomitant with suppressed expression of cyclin D1 (cell cycle progression-promoting protein) (40, 98). Hence, targeted methods based on integrin-α9β1 or fibronectin-EDA can serve as treatment modes for the inhibition of cancer progression and limitation of colon cancer progenitor cells.

#### Rhabdomyosarcoma

Rhabdomyosarcoma is an early onset malignancy, which is the most common type of soft tissue sarcoma in children. About 15% of rhabdomyosarcoma patients are diagnosed with distant metastasis (99, 100). Despite the prognosis of most patients with field cancerization being acceptable, the effectiveness of treatments for metastatic rhabdomyosarcoma is particularly poor and the long-term event-free survival rate is less than 20% (101). Integrin-α9β1 has been demonstrated as directly involved in the attachment, migration and invasiveness of rhabdomyosarcoma cells, and the specific anti-α9-blocking antibodies significantly decrease the invasive properties (74).

The Notch pathway plays an important role in the expressions of *ITGA9* and N-cadherin, for which the downstream effectors Hes1 and NICD directly bind to their promoter regions. This mechanism may explain why the pharmacological Notch signaling inhibitor (GSI) impairs the adhesion of rhabdomyosarcoma cells. For the purpose of metastasis resists, Notch-inhibiting molecules serve as potential therapeutic agents (74). Moreover, after a screening process, a recent analysis selected miR-7 and miR-324-5p as the best candidates for regulating integrin-α9β1. Overexpression of both types of miRNA leads to genetic down-regulation of *ITGA9* following sharp decreases in cell proliferation and invasion *in vitro* and *in vivo*, since integrin-α9β1 participates in cell proliferation and tumour growth in the two main rhabdomyosarcoma subtype cell lines RD and CW9019. In the murine model with intravenously injected tumour cells, miR-7 also induces significant impairment of rhabdomyosarcoma cell metastatic lung colonization (75). This raises the possibility of using *ITGA9*-mediating miRNA as a novel therapeutic tool to avoid rhabdomyosarcoma progression.

#### Squamous Cell Carcinoma (SCC)

In the healthy oral mucosa, integrin-α9β1 is mainly expressed at the basal and immediately suprabasal cell layers. However, in leukoplakic dysplasia, lichen planus and SCC samples, α9β1 is more diffusely expressed at the epithelial cell membranes (102), since both leucoplakia and lichen planus are considered to be potential oral malignant disorders with certain risks of malignant transformation (103). TNC is reported to be a promoter of pro-tumorigenic microenvironments as a classical ligand for integrin-α9β1, taking part in immune suppression in oral SCC (76). It works with α9β1 and TLR4 to activate the CCL21/CCR7 signaling pathway and induce the expression of CCL21, which is known to be a chemoattractant for various leukocytes and lymphoid tissue. This shifts the host’s immunogenic immune response to being a tolerogenic response and facilitates tumour progression and metastasis by evading immune surveillance (104). Either integrin-α9β1 or TNC antibodies reduce CCL21 mRNA and protein expression to improve hypoimmunity, suggesting that blocking integrin-α9β1 or TNC can alter the SCC tumour microenvironment (76).

#### Hepatic Fibrosis and Hepatocellular Carcinoma

Integrin-α9 chains can be detected in hepatocytes and fetal hepatoblasts, and the latter likely performing a key role in the differentiation of liver stem cells (105). Thrombin-cleaved OPN (which exhibits the α9-binding motif) interacts with α9β1 to promote activation, proliferation and migration of hepatic stellate cells *via* the mitogen-activated protein kinase (MAPK) and nuclear factor-kappa B (NF-κB) signalling pathway, which are essential for liver fibrogenesis (52). In addition, integrin-α9β1 is also important for activating the motility of hepatic stellate cells through cooperation with ligand fibronectin-EDA (106). The sustained exacerbation of liver fibrosis predisposes certain individuals to cirrhosis or even hepatocellular carcinoma (107). However, it is interesting that unlike most other types of tumors, the expression level of *ITGA9* obviously declines in hepatocellular carcinoma and works as an inhibitor of the migration and invasion of hepatocellular carcinoma cells *via* the FAK/Src (c-Src tyrosine kinase)-Rho GTPases signalling pathway (77). *ITGA9* overexpression inactivates the Rho GTPases members Rac1/RhoA and reduces FAK/Src phosphorylation, which are important in tumour angiogenesis and protease-associated metastasis (108, 109). Although the protein or mRNA of *ITGA9* are roughly increased in the above-mentioned cancer types, they have previously been reported to be down-regulated in other tumors, such as human squamous cell carcinoma of the head and neck, bladder cancer and non-small-cell lung cancer, which is similar to the case for hepatocellular carcinoma (110–112). The research reviewed above reveals that ITGA9 has the potential to become a diagnostic biomarker for hepatocellular carcinoma, provides a potential treatment solution and, more importantly, shows notable tumour heterogeneity in different cancer types.

### Integrin-α9β1 Serves as a New Target for the Treatment of Autoimmune Diseases

**Figure 2:**
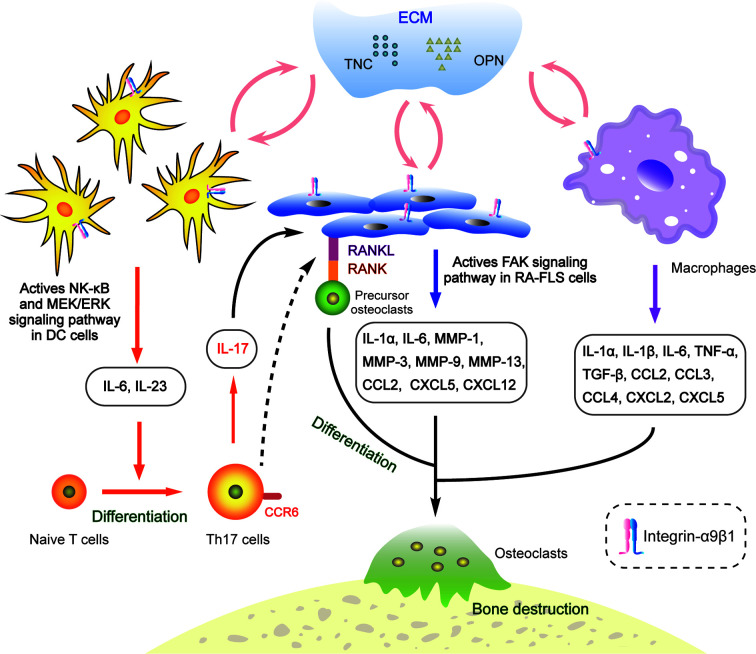
Interactions between integrin-α9β1 and the extracellular matrix (ECM) ligands TNC and OPN regulate the RA microenvironment. OPN and TNC bind to α9β1, promoting secretion of inflammation-related factors in conventional dendritic cells, synovial fibroblasts and macrophages, subsequently inducing osteoclast differentiation and inflammatory cell infiltration and, ultimately, leading to bone destruction.

In the last decade, more and more literature has focused on the characteristics of integrin-α9β1 in autoimmune diseases. It has been identified as a novel putative therapeutic target for rheumatoid arthritis [RA; (113)]. Various extracellular matrix proteins, like TNC and OPN, have been demonstrated to up-regulate at the hyperplasia of joint synovium in RA (114, 115). They act as ligands for integrin-α9β1 to transduce intracellular mechanical signals (116) and are deeply involved in the arthritic inflammatory microenvironment (**Figure 2**).

*ITGA9* is overexpressed in human RA samples and precedes the onset of arthritic symptoms in experimental models (113). The membranes of both synovial fibroblasts and synovial macrophages contain integrin-α9β1 but contribute differently to the production of various chemokines that are responsible for the recruitment and activation of inflammatory cells (117). In synovial fibroblasts, TNC and OPN interact with α9β1 to induce expressions of IL-1α, IL-6, MMP-1, -3, -9, -13, CCL2, CXCL5 and CXCL12. Meanwhile the expression levels of IL-1α, IL-6, TNF-α, IL-1β, TGF-β, CCL2, CCL3, CCL4, CXCL2 and CXCL5 increase in synovial macrophages (117, 118). Furthermore, in the conventional dendritic cells of an arthritis rodent model, integrin-α9β1 collaborates with TNC and OPN to induce secretion of IL-6 and IL-23 through both the NF-κB and MEK/ERK signalling pathways (**Figure 2**), which promotes the generation of pathogenic Th17 cells involved in osteoclast differentiation and bone destruction (119). Blocking of α9β1 sharply inhibits the release of these chemokines, which contribute to inflammatory cell activation and recruitment as well as angiogenesis and osteoclastogenesis (118). Therefore, integrin-α9β1 and its ligands act as key intrinsic mediators in the arthritis process (120–122).

In an arthritis animal model with inducements of anti-type II collagen antibody and lipopolysaccharide (123), injection of special anti-integrin-α9 (55A2C) antibody clearly reduced the number of arthrogenic cytokines and chemokines and ameliorated ongoing arthritis, demonstrating the therapeutic potential of the anti-α9 antibody for RA (117). Similarity, another study reported that intraperitoneal injection of blocking α9 antibody MA9-413 in collagen-induced arthritic mice also repressed the development of arthritis and inflammatory cell infiltration (113). Concurrently, it significantly decreased activated fibroblast-like synoviocyte (FLS)-derived biomarkers like MMP-3, IL-6, sRANKL and CXCL5 in plasma (124–126). MA9-413, also known as AS2535093, specifically recognizes a conserved loop region designated as amino acids 104–122 of α9, which binds to integrin-α9 but not α4 in humans and mice. In bone marrow cells of arthritic mice, MA9-413 considerably decreases RANKL and IL-6 expressions while inhibiting osteogenic differentiation (127). More important, this α9 antibody does not influence the number of immune cells in the spleen when it significantly prevents lymphocyte accumulation in the arthritic joint microenvironment, so MA9-413 plays a therapeutic role with minimal systemic immunomodulation (113).

Another study has demonstrated that the disruption of α9 with shRNA also inhibits the expression of TNC because of a positive feedback loop in which the production of TNC is dependent on α9β1-dependent FAK activation of integrin signalling (45). Compared to osteoarthritis, the FLSs of RA have activated FAK-mediated integrin-α9 signalling. Knockdown of either *ITGA9* or *TNC* in RA-FLSs quells the phosphorylation of FAK, which is crucial for RA-FLS cell adhesion, migration and intrinsic secretion of proinflammatory mediators. Disruption of α9β1 or TNC also inhibits the spontaneous creation of MMP1, MMP3, MMP14, IL-6, cadherin-11 and TNFSF11/RANKL. Moreover, transfection of shRNA-ITGA9 in 3D cultured RA-FLSs abrogates abnormally condensed cellular accumulation structures (which reflects a pathogenic feature) and shows no proliferative reaction to stimulation of platelet-derived growth factor. Meanwhile, the production of proinflammatory regulators in response to exogenous TNF-α is nearly entirely absent compared with controls (45, 128). These results suggest that integrin-α9β1 plays an essential role in the aggressive behavior of RA-FLSs, both under autonomy conditions and exogenous inflammatory stimuli.

As mentioned above, OPN is also a key molecule in arthritis, since it binds to integrin-α9β1 *via* the SVVYGLR amino acid sequence rather than the full-length sequence (129). The thrombin cleaves OPN, accompanied by subsequent exposing of the SVVYGLR sequence at the N-terminal fragment in humans (130). However, in rats and mice, the amino acid sequence reacting to α9β1 is replaced by SLAYGLR (131). Intravenous injection of the specific antibody reacting to the SLAYGLR sequence inhibits the proliferation of synovium, inflammatory cell infiltration and bone erosion in murine arthritic joints (132), demonstrating that the cryptic epitope is crucial to RA pathogenesis. Deferring from thrombin, MMP-3/7 cleaves mouse OPN to expose the new sequence LRSKSRSFQVSDEQY at the C-terminal fragment, which also binds to α9β1 to participate in anti-type II collagen antibody-induced arthritis. Meanwhile, the antibody of the LRSKSRSFQVSDEQY sequence also shows an ability to diminish the pathological features of arthritis. These results indicate that MMP-3 and MMP-7 promote the development of RA *via* interaction between OPN and integrin-α9β1 (133).

Recently, TNC, OPN and common receptor integrin-α9β1 have been identified as promising treatment targets for more autoimmune inflammatory diseases, since both TNC- and OPN-deficient mice have shown resistance against a variety of Th1- and/or Th17-related autoimmune diseases (134). Except for RA, integrin-α9β1 is also involved in experimental autoimmune encephalomyelitis in mice *via* regulation of the secretion of sphingosine-1-phosphate (S1P) receptors, which affect the discharge of immune cells and are related to chronic periodontitis *via* the MAPK pathway (116, 135). It is remarkable that the new integrin-α9 ligand XCL1/lymphotactin has also been reported to participate in RA and autoimmune encephalomyelitis. Both α9β1-neutralizing and XCL1-neutralizing antibodies ameliorate the symptoms of autoimmune disease in murine models, and injection of α9β1-antibody relieves experimental autoimmune encephalomyelitis-related symptoms with clear reductions in spinal cord-infiltrating cells and lymphocyte egress *via* lymph node drainage (56, 136).

It is noteworthy that, apart from regulating lymphocyte egress *via* lymphatic endothelial cells, integrin-α9β1 is also essential for lymphatic valve formation and maintenance (32), which are closely related to the rejection of transplantation (137). Subconjunctival injection of α9-antibodies during orthotopic keratoplasty in mice can inhibit the formation of lymphatic valves without the intervention of lymphangiogenesis, so as to significantly improve the survival rate of grafts after transplantation (138, 139).

In conclusion, local antibody neutralization therapy using integrin-α9β1 or corresponding ligands may be prospective therapeutic directions for treating various refractory immune diseases, even for preventing immune rejection of transplanted organs. Hence, the underlying mechanism of integrin-α9β1 participation in immune-related cellular functions is worthy of further research and has valuable clinical significance.

### Integrin-α9β1 Promotes Axon Regeneration

After neuronal injury of the central nervous system (CNS), it is difficult for axons to regenerate and recover function, due to the incompetent intrinsic regenerative ability of adult CNS neurons and inhibitory factors in the microenvironment (140). Axonal growth is a particular form of cell migration; meanwhile, integrins and ligands are crucial in cell adhesion and neuronal migration (141), so inhibitors of integrins that are present in the microenvironment are non-negligible factors in blocking CNS regeneration (142). For example, the integrin response suppressors, myelin-derived Nogo-A protein and chondroitin sulfate proteoglycans (CSPGs), impair integrin signalling by decreasing phosphorylated FAK and Src levels, which may be a potential factor affecting nerve self-repair (143, 144).

Furthermore, during postnatal corticospinal axon development, cortical neurons introduce integrins into their axons. However, integrins are clearly excluded from the axons when the cortical neurons mature, especially the key receptor integrin-α9β1, which is considered to be an important reason for the low regenerative competence of the CNS (145). After damage to the CNS, TNC is up-regulated, which is the main extracellular matrix glycoprotein in the CNS environment and the important ligand of integrin-α9β1; however, the α9 subunit is absent in adult neurons. Forced expression of α9 leads to neurite outgrowth in both PC12 cells and dorsal root ganglia axon (DRG) neurons of adult rats (46), suggesting that the reaction between α9β1 and TNC might play a key role in axonal regeneration. Kindlin-1 is reported to counteract the effects of CSPGs and Nogo-A to enhance integrin activation and signalling in the DRG of rats and promote axon growth with sensory axon regeneration (146). The interaction between kindlin-1-activated integrin-α9β1 and TNC overcomes the inhibitory environment of the adult axons: overexpression of kindlin-1 and *ITGA9* can achieve long-distance sensory axon regeneration (> 25 mm axon length) and sensory–motor recovery in rats, which has great clinical significance, while overexpression of α9 or kindlin-1 alone is associated with substantially lower recovery and regeneration (147).

In general, it is difficult to effectively transport integrin-α9 into CNS axons, since it is restricted to being transported along axons in mature cortical neurons (148). The small GTPase Rab11 regulates the key pathway of integrin transport and participates in the transport of various neuron membrane molecules (149–151). It has been shown that α9 can be vesicle-transported through Rab11 and RCP (Rab11 effector Rab coupling protein) in differentiated PC12 cells and adult DRG neurons (152), revealing that manipulation of Rab11 and RCP may be beneficial to neuron therapy after injury. However, the transportation speed of integrins through Rab11 is not fast, and another study showed that the rapid transport of axons and cell surfaces is regulated by ARF6, which is involved in the exclusion of integrins from mature CNS axons (153). The ARF6 inactivator ACAP1 (ARF6 GTPase-activating protein) increases axonal growth, integrin-α9 externalization and anterograde transport, while the ARF6 activator EFA6 and ARNO (neuronal ARF6 guanine nucleotide exchange factors) suppress axon growth with increases in integrin retrograde transportation and internalization in the DRG of adult rats (154). Therefore, the role of ARF6 inhibitors in nerve regeneration is worthy of further exploration.

On the other hand, transplantation of human-induced pluripotent stem cell-derived neural progenitor cells (NPCs) is considered to be another potential regenerative therapy after nervous system injury, since hNPCs produce endogenous α9 and β1 subunits (155). Both wild-type and lentivirus-mediated overexpressing-α9 hNPCs induce axonal growth in the developing nervous system of rats, but their effects on spinal cord injury remain to be studied. Besides, integrin-α9β1 and TNC synergistically improve the efficiency of differentiation from mesenchymal stem cells into neuronal lineages, which has important implications for stem cell therapies (156).

In brief, integrin-α9β1 has a significant supportive effect on recovery and regeneration after nerve injury but is absent and suppressed in adult neurons. The manipulation of increased α9 expression, transport and activation, could become valuable strategies for driving integrin-dependent axonal regeneration.

### Integrin-α9β1 Makes a Promising Target for Antithrombotic Therapy

Integrin-α9β1 has attracted attention as a potential new target for antithrombotic therapy in recent years (157). Numerous studies have noted the importance of neutrophils in thrombosis formation *via* modulation of platelet adhesion, activation and coagulation, as well as by facilitating coordinated interaction between endothelial cells and platelets (158–160). Compared to monocytes, integrin-α9β1 is highly expressed in neutrophils and is essentially for neutrophil development, since *ITGA9*-deficient mice have dramatic defects in neutrophil production due to a decrease in bone marrow granulocyte precursors, accompanied by a reduced capacity to differentiate bone marrow cells into granulocytes (18, 49). Besides, α9β1 is expressed on polymorphonuclear leukocytes in human blood and is up-regulated after leukocyte activation, implying its potential role in neutrophil migration through lung and synovial fibroblast barriers (161). For instance, in aspirated pneumonia patients, the expression of α9β1 in circulating neutrophils is significantly higher than that in healthy people, indicating that integrin-α9β1 may play a potential role in neutrophil extravasation (162).

**Figure 3:**
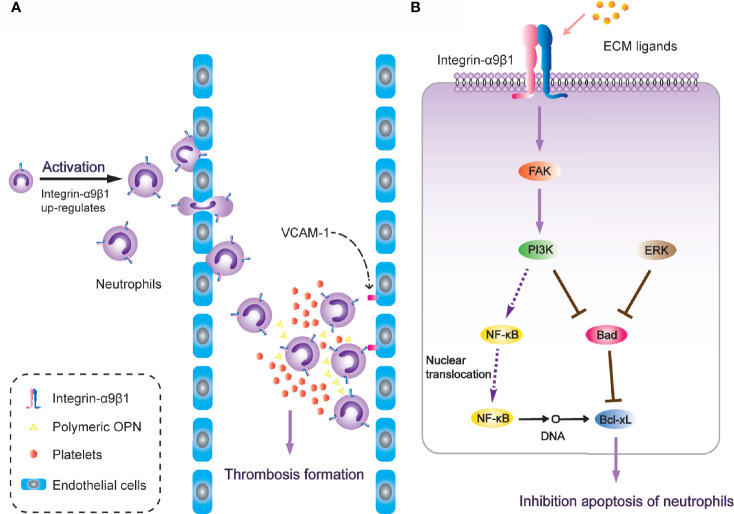
Integrin-α9β1 is involved in thrombosis through the regulation of chemotaxis, adhesion and apoptosis of neutrophils. **(A)** Integrin-α9β1 up-regulates during neutrophil activation and interacts with its ligands (VCAM-1 and Polymeric OPN), mediating neutrophil chemotactic activity and stabilizing adhesion to endothelial cells, eventually resulting in increased risk of thrombosis. **(B)** Integrin-α9β1 inhibits apoptosis of neutrophils through the PI3K and ERK signaling pathways.

It is worth noting that α9β1 regulates the physiological activity of neutrophils through a variety of different ligands. After neutrophil activation, the expression of α9β1 has been detected to increase two-to-three-fold. During neutrophil transendothelial migration through human umbilical vein endothelial (HUVE) cell-coated transwells, α9β1 was the only up-regulated β1-type integrin (163). Antibodies against either α9β1 or VCAM-1(vascular cell adhesion molecule-1) down-regulate the augmented migration across TNF-α-activated HUVE cell monolayers (49), and VCAM-1 is also a fundamental ligand for α9β1 in regulating cell adhesion [**Figure 3A**; (50)]. Additionally, after release from bone marrow, neutrophils undergo spontaneous apoptosis within 24 hours under normal physiological conditions (164). However, in inflammatory tissues, the survival of neutrophils is significantly prolonged, while the interaction between VCAM-1 and α9β1 involves inhibition of neutrophil apoptosis through PI3K and NF-κB activation (165). This induces an extension of lifespan upon full activation of the neutrophils, so as to promote thrombosis (166). Another study revealed that integrin-α9β1 activates the PI3K and MAPK-ERK signalling pathways in human neutrophils with NF-κB nuclear translocation, pro-apoptotic protein Bad degradation and enhanced anti-apoptotic protein Bcl-xL, resulting in spontaneous delay of cell apoptosis [**Figure 3B**; (167)]. The novel heterodimeric disintegrins EC3 and EC6, which have been isolated from the venom of *Echis carinatus*, are both effective inhibitors of adhesion mediated by reaction between integrin-α9β1 and VCAM-1. They also disrupt neutrophil migration across endothelial cells. These natural integrin inhibitors are considered to have a therapeutic potential to inhibit excessive migration of leukocytes through integrin-α9β1 (168).

Polymeric-OPN, which is another ligand that employs integrin-α9β1 as the receptor and is formed by transglutaminase mediation, can attract neutrophils by presenting a special binding site, while unpolymerized OPN cannot (169). Polymeric-OPN has been detected in aortic tissue and bone and induces neutrophil recruitment *via* α9β1 in a mouse model, in which injection of the transglutaminase inhibitor cystamine attenuates the recruitment (53). Furthermore, OPN has also been reported to interact with both integrin-α4β1 and α9β1 in neutrophils in an alcoholic liver disease rodent model, causing high hepatic neutrophil infiltration and liver injury (170). Interestingly, the reaction sites of OPN exposure reported by these studies are diverse. The latter declared that the SVVYGLR fragment of thrombin-cleaved OPN was identified by integrin-α9β1 to induce neutrophil infiltration; however, the former reported that the polymeric-OPN did not have this classical sequence, suggesting that OPN might present an undiscovered binding site after transglutaminase mediation. The same point is that α9β1 cannot recognize the complete OPN, which needs proper enzyme treatment to play the role of a ligand. Furthermore, different enzymes produce divergent binding sites, suggesting a redundant mechanism of OPN and α9β1 in neutrophil chemotaxis.

ADAM family members selectively modulate different integrin-mediated cell migrations as extracellular matrix ligands (171). ADAM9D has been proven to contribute to neutrophil activation and chemotaxis *via* the cooperation of integrin-α9β1 and αVβ3, concomitant with activation of the PI3K/Akt and ERK pathways, while blockade of either α9β1 or αVβ3 impairs the migration of human neutrophils toward ADAM9D (172). The PI3K/Akt pathway is involved in leukocyte function and the recruitment of both neutrophils and macrophages (173). It also leads to subsequent phosphorylation of the ERK, which supports the antiapoptotic function of integrin-α9β1 for neutrophils [**Figure 3B**; (167)].

It has been reported that myeloid cell-specific integrin-α9^-/-^ mice that were less susceptible to arterial thrombosis and had unaltered hemostasis under conditions of ferric chloride and laser injury-induced thrombosis. They had reduced numbers of neutrophils, red blood cells and myeloperoxidase levels in the diminished carotid thrombi compared with normal mice. More striking was the therapy of a wild-type group with anti-α9 mAb (55A2C), which obviously suppressed ferric chloride-induced arterial thrombosis, thereby revealing the suitability of α9 as a therapeutic target for arterial thrombosis (174). It is also worth noting that deletion of the *ITGA9* gene from myeloid cells can improve both short- and long-term stroke outcomes and survival rates in an ischemic stroke rodent model. This is concomitant with a reduction in the cerebral thrombo-inflammatory response, as evidenced by decreases in fibrin, platelet thrombi, neutrophils, phospho-NF-κB, TNF-α and IL-1β levels, as well as diminishment of neutrophil extracellular trap formation (web-like chromatin structures that induce activation of endothelial cells, antigen-presenting cells and platelets, as well as triggering the proinflammatory immune response, atherosclerotic plaque formation and arterial thrombosis). In addition, intravenous infusion of 55A2C antibody into hyperlipidemic mice following reperfusion significantly reduces infarct volume and improves both short-term and long-term functional outcomes (175, 176). Taken together, these studies show that the targeting of myeloid-specific integrin-α9β1 may become a new treatment direction for thrombotic diseases.

## Discussion and Future Prospects

In this paper, we provide an enhanced and updated review of current research on integrin-α9β1 as a therapeutic target for different refractory diseases, focusing on the trends and changes that have occurred in the past ten years. As mentioned above, the specific antibodies, microRNA and other inhibitors that target integrin-α9β1 or corresponding ligands have shown therapeutic effects on tumors, autoimmune diseases and thrombosis. On the other hand, the overexpression, transport and activation of integrin-α9β1 hold great promise in curing axon damage.

The function of α9β1 is mainly driven by corresponding ligands in the extracellular matrix. These form a complicated signalling network and regulate the physiological and pathological behaviors of cells. However, the reactions are redundant and complex, because co-existing ligands simultaneously collaborate with integrin-α9β1 to mediate the same or different signalling pathways. Although the experiments considered in this review testify to the effectiveness of blocking these ligands to interdict the pathological process, other additional effects were not fully considered. This is because a large fraction of ligands not only interact with α9β1 but also react to other receptors and create crosstalk of molecular signalling pathways in a variety of ways (177–179). On the other hand, despite the functional alternation of α9β1 having been demonstrated to have therapeutic potential in many animal models, the possible side-effects remain to be studied. Since integrin-α9β1 also plays an indispensable role in normal physiological processes, such as the development and renewal of lymphatic and venous valves (19, 41) and proper re-epithelialization in cutaneous wound healing (180). We were pleasantly surprised to discover a very recent clinical trial using anti-α9 antibodies. ASP5094, a humanized mAb against integrin-α9, was used in a phase 2a, multicenter, randomized, double-blind study to cure refractory RA with resistance to methotrexate (181). Although intravenous ASP5094 (10 mg/kg) did not show efficacy in patients with moderate to severe refractory RA, this result could be due to insufficient exposure of ASP5094 in the target tissue. No safety signals were evident, such that ASP5094 is considered to be well-tolerated and safe overall. Because integrin-α9β1 is positioned at the cell membrane, local injection of inhibitors can act as an effective blocking factor in lesion locations (182) and play a therapeutic role through non-immunosuppressive pathways, which may benefit treatment without excessive systemic effects (45). Consequently, we look forward to further clinical trials that target α9β1 with diverse treatment modalities.

However, notwithstanding that integrin-α9β1 is distributed in so many types of organs and cells, its wider range of activity and precise mechanisms require further investigation, given that some recognized α9β1 ligands are up-regulated in many diseases. For example, TNC is remarkably increased in bronchoalveolar lavage fluid and serum in coronavirus disease 2019 (COVID-19) patients, while serum levels of VCAM-1 are also elevated in mild cases and dramatically up-regulated in patients with severe disease (183, 184). Likewise, VEGF has been reported to be involved in the brain inflammation caused by attack from severe acute respiratory syndrome coronavirus 2 (SARS-COV-2, the viral pathogen of COVID-19) (185). These extracellular matrix molecules are considered to be biomarkers or therapeutic candidates for COVID-19 and are under a recognized ligand of α9β1. For this reason, it can be inferred that α9β1 may play a regulatory role in the pathological process of COVID-19. In addition, OPN is highly associated with autoimmune diseases of the skin, such as lupus erythematosus and pemphigus vulgaris (186, 187); hence, it is likely to work with the α9β1 in skin cells to produce inflammatory reactions similar to RA, as previously described. Overall, α9β1 is a potential mediator of other diseases and further insights are urgently needed.

To summarize, treatments targeting integrin-α9β1 have been effective in many experiments and α9β1 may play important roles in more unexplored diseases. Integrin-α9β1 is of great research value as a candidate therapeutic target for clinical treatment and its future prospects are worth exploring.

## Author Contributions

SX, TZ, WZ, CZ and HL collected the data. SX wrote the article. ZC and JS reviewed the article. All authors contributed to the article and approved the submitted version.

## Funding

This work was supported by the National Natural Science Foundation of China (Grant Nos. 81771082, 31971282, 81800985) and the Chongqing Graduate Tutor Team (2019).

## Conflict of Interest

The authors declare that the research was conducted in the absence of any commercial or financial relationships that could be construed as a potential conflict of interest.
